# Current trends in optimal medical therapy after PCI and its influence on clinical outcomes in China

**DOI:** 10.1186/s12872-021-02052-z

**Published:** 2021-05-26

**Authors:** Jian Zhang, Jing-Yan Hao, Rui Jing, Jing-Jing Liu, Cheng-Ye Di, Yu-Jie Lu, Peng Gao, Ya-Jie Wang, Rui-Fei Yang, Wen-Hua Lin

**Affiliations:** 1 Department of Cardiology I, TEDA International Cardiovascular Hospital, Tianjin, 300457 China; 2 College of Clinical Cardiovascular Disease, Tianjin Medical University, Tianjin, China

**Keywords:** Coronary heart disease, Optimal medical therapy, Post-PCI, Prognosis, Predictors

## Abstract

**Background:**

Limited data were available on the current trends in optimal medical therapy (OMT) after PCI and its influence on clinical outcomes in China. We aimed to evaluate the utilization and impact of OMT on the main adverse cardiovascular and cerebrovascular events (MACCEs) in post-PCI patients and analyzed the factors predictive of OMT after discharge.

**Methods:**

We collected data from 3812 individuals from 2016.10 to 2017.09 at TEDA International Cardiovascular Hospital. They were classified into an OMT group and a non-OMT group according to their OMT status, which was defined as the combination of dual antiplatelet therapy, statins, β-blockers, angiotensin-converting enzyme inhibitors or angiotensin receptor blockers after PCI. Multivariable Cox regression models were developed to assess the association between OMT and MACCEs, defined as all-cause mortality, nonfatal myocardial infarction, stroke, and target vessel revascularization. A logistic regression model was established to analyze the factors predictive of OMT.

**Results:**

Our results revealed that the proportion of patients receiving OMT and its component drugs decreased over time. A total of 36.0% of patients were still adherent to OMT at the end of follow-up. Binary logistic regression analysis revealed that baseline OMT (*P* < 0.001, *OR* = 52.868) was the strongest predictor of OMT after PCI. The Cox hazard model suggested that smoking after PCI was associated with the 1-year risk of MACCE (*P* = 0.001, *HR* = 2.060, 95% CI 1.346–3.151), while OMT (*P* = 0.001, *HR* = 0.486, 95% CI 0.312–0.756) was an independent protective factor against postoperative MACCEs.

**Conclusions:**

There was still a gap between OMT utilization after PCI and the recommendations in the evidence-based guidelines. Sociodemographic and clinical factors influence the application of OMT. The management of OMT and smoking cessation after PCI should be emphasized.

## Background

Coronary heart disease (CHD) remains the most significant cause of mortality and a substantial contributor to reduced quality of life worldwide [1, 2]. Along with improved stent design, procedural techniques, and effective pharmacological treatments, revascularization therapy has become an essential part of the treatment of CHD [3, 4]. Nonetheless, cardiovascular and non-cardiovascular death and other ischemic events driven by atherosclerosis after percutaneous coronary intervention (PCI) remain concerning [5]. Cardiovascular death is the predominant issue in the short term after PCI [6]. The progression of atherosclerosis does not stop, even in patients who have undergone coronary revascularization with PCI or coronary artery bypass grafting (CABG) [7]. Mechanical manipulation can also cause damage to blood vessels, which can lead to the occurrence of adverse cardiovascular events. Although revascularization with PCI or CABG has repeatedly demonstrated to be the best treatment for patients with severe coronary stenosis or occlusion, intraoperative or perioperative medical therapy is generally emphasized. In contrast, concomitant optimal medical therapy (OMT) is often ignored after patients have undergone PCI.

OMT is recommended in the current practice guidelines to improve further symptoms and reduce the incidence of adverse events [8, 9]. In recent years, several registries have evaluated the use of OMT after PCI. There are substantial within-country differences in the management of patients after PCI [10], as is the case in China. Moreover, several observational studies found that the application of OMT after PCI was suboptimal in the real world [11, 12]. Limited data are available on the use of OMT after PCI in China.

Accordingly, our study aimed to investigate the long-term trend in the utilization of OMT, evaluate the utilization and impact of OMT on the main adverse cardiovascular and cerebrovascular events (MACCEs) in post-PCI patients and analyze the predictive factors of OMT after patient discharge in China.

## Methods

### Patient enrollment

**Fig. 1:**
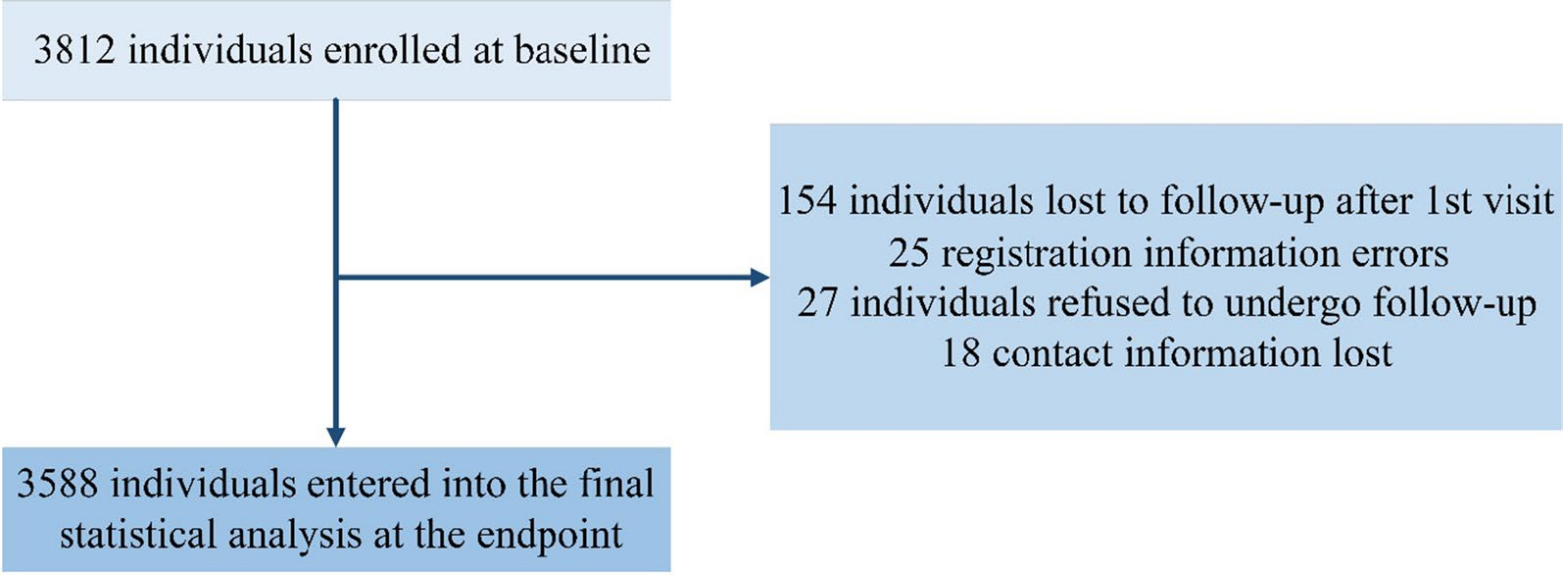
Study flowchart

Our study was a single-center prospective observational study. A total of 3812 patients diagnosed with coronary heart disease who successfully underwent PCI at TEDA International Cardiovascular Hospital from 2016.10 to 2017.09 were selected. The study protocol was approved by the TEDA International Cardiovascular Hospital Research Ethics Committee, and informed consent was obtained from the patients before enrollment. Figure 1 showed the study flow chart. The inclusion criterion was successful PCI. The following were exclusion criterion: (1) a definite history of allergy and allergies to or intolerance of the drugs recommended by the guidelines (including statins, antiplatelet drugs, β-blockers, and various blood pressure and glucose-lowering drugs); (2) a diagnosis of malignant tumors or a life expectancy < 1 year; (3) a diagnosis of an immune system disease and/or taking hormones therapy; (4) a serum creatinine level ≥ 265 mol/L or renal failure detected in the past or during hospitalization; (5) lack of autonomy or a diagnosis of a mental illness; (6) incomplete clinical data or coronary angiography data; and (7) in-hospital death after PCI.

### Data collection

Demographic data included age, sex, height, weight, occupation, education, and type of medical insurance. Other clinical data included admission diagnosis, family history of cardiovascular disease and history of hypertension, diabetes, hyperlipidemia, chronic heart failure (CHF), chronic renal insufficiency, stroke, old myocardial infarction, angina pectoris, PCI, CABG, smoking and other concomitant diseases. We also collected data from all included patients on the levels of hemoglobin, low-density lipoprotein cholesterol, fasting blood glucose, serum creatinine, alanine aminotransferase and the surgical records, including information about the vascular lesions and the number of stents placed. All clinical data were obtained from the electronic medical record system.

### Follow-up, definitions, endpoints

In this study, medication use was recorded before discharge and at 1 month, 6 months and 1 year after discharge. MACCEs and the occurrence of individual events were also recorded. Follow-up was mainly performed by telephone (contact with the patient or the patient’s family), outpatient clinic visits and readmission. OMT was defined as the combination of dual antiplatelet therapy (DAPT), statins, β-blockers, and angiotensin-converting enzyme inhibitors (ACEIs) or angiotensin receptor blockers (ARBs) during follow-up after PCI. The primary endpoint was the occurrence of any MACCEs, defined as a composite of death from any cause, nonfatal myocardial infarction, stroke, or target vessel revascularization (TVR). The secondary endpoints were each of the individual clinical events described above. TVR was defined as repeat PCI or CABG in the vessel treated during the index PCI or any coronary artery segment containing the target lesion.

### Statistical analysis

All statistical analyses were performed with SPSS 22.0 (SPSS Inc., Chicago, Illinois). Continuous variables with a normal distribution are expressed as the means ± standard deviations (`x ± s). Independent samples t-tests were used to compare the differences between groups. Nonnormally distributed data are represented as the median (quartile ranges) [M(Q1–Q3)]. The Mann–Whitney U test was used for intergroup comparisons. Logistic regression was used to calculate the odds ratio (OR) with 95% confidence intervals (CI) to find out the factors on OMT status after PCI. OMT status was regarded as the dependent variable. Socio-demographic and medical variables were independent variables. Chi-square test was used to compare the categorical variables between groups. The effect of baseline variables on OMT status was equalized by applying propensity scores match (1:1 match, caliper value set to 0.02). Multivariable Cox regression model was developed to calculate the hazard ratio (HR) with 95% CI for significant differences and clinically significant variables between groups on MACCEs. MACCEs status were regarded as the dependent variable. Significant differences and clinically significant variables between groups were independent variables. *P *value below 0.05 was considered statistically significant. The follow-up period was from admission to the day when the MACCE was observed or until 1 year after discharge from the hospital.

## Results

### Baseline data analysis

Our study included 3812 patients who underwent PCI at TEDA International Cardiovascular Hospital from 2016.10 to 2017.09. The median follow-up was 13.0 months (range from 0.2 to 13.0); 224 patients were lost to follow-up (including those who refused follow-up, changed contact number or stopped responding), and 3588 patients were included in the statistical analysis (Fig. 1). As shown in Table 1, the study population was dominated by male ACS patients with high prevalence of smoking (62.2%) and hypertension (64.2%). Approximately 30% of patients had a myocardial infarction or revascularization, and 89.0% were diagnosed with ACS (ST-elevation myocardial infarction, non-ST-elevation myocardial infarction, or unstable angina). Patients treated with OMT at the end of follow-up had higher incidence of baseline coronary risk factors (e.g., older age, history of hypertension, hyperlipidemia, diabetes), more comorbidities and more advanced coronary artery disease (as measured by the number of diseased vessels). See Table 1 for details.

**Table 1:** Baseline data of patients grouped by OMT status

	Total (n = 3588)	OMT (n = 1299)	Non-OMT (n = 2289)	*P* value
*Demographics*				
Age (years)	62.20 ± 9.72	62.69 ± 9.40	61.93 ± 9.89	0.025
≥ 65 years	1479 (41.2)	560 (43.1)	919 (40.1)	0.083
Male	2298 (64.0)	815 (62.7)	1483 (64.8)	0.219
BMI (kg/m^2^)	25.74 ± 3.23	25.93 ± 3.27	25.63 ± 3.20	0.009
Medical insurance				< 0.001
No medical insurance	609 (17.0)	132 (10.2)	477 (20.8)	
Basic medical insurance	1411 (39.3)	436 (33.6)	975 (42.6)	
Employees medical insurance	1568 (43.7)	731 (56.3)	837 (36.6)	
Education				< 0.001
Primary	1088 (30.3)	334 (25.7)	754 (32.9)	
Intermediate	1060 (29.5)	310 (23.9)	750 (32.8)	
Advanced	1440 (40.1)	655 (50.4)	785 (34.3)	
Current smoker	1444 (40.2)	537 (41.3)	907 (39.6)	0.314
*History*				
Prior PCI	878 (24.5)	347 (26.7)	531 (23.2)	0.019
Prior CABG	54 (1.5)	21 (1.6)	32 (1.4)	0.602
Prior MI	475 (13.2)	185 (14.2)	290 (12.7)	0.182
*Comorbidities*				
Hypertension	2305 (64.2)	1023 (78.8)	1282 (56.0)	< 0.001
Hyperlipidemia	1494 (41.6)	591 (45.5)	903 (39.4)	< 0.001
Diabetes	1828 (50.9)	724 (55.7)	1104 (48.2)	< 0.001
Stroke	287 (8.3)	117 (9.0)	180 (7.9)	0.232
CHF	249 (6.9)	80 (6.2)	169 (7.4)	0.165
Number				< 0.001
0	416 (11.6)	86 (6.6)	330 (14.4)	
1	1021 (28.5)	313 (24.1)	708 (30.9)	
≥ 2	2151 (59.9)	900 (69.3)	1251 (54.7)	
*Diagnosis*				
ACS	3195 (89.0)	1160 (89.3)	2035 (88.9)	
Number of coronary lesions				0.003
Single	1013 (28.2)	328 (25.3)	685 (29.9)	
Multiple (≥ 2 branches or left main obstructive)	2575 (71.8)	971 (74.7)	1604 (70.1)	
PLT (10^9/L)	223.38 ± 55.89	224.26 ± 56.60	222.88 ± 55.49	0.479
HGB (g/L)	136.69 ± 15.42	136.61 ± 15.42	136.73 ± 15.42	0.822
FBG (mmol/L)	7.60 ± 2.76	7.83 ± 2.79	7.48 ± 2.74	< 0.001
SCR (µmol/L)^a^	67(57,78)	68(58,80)	67(57,77)	0.016
LDL-C (mmol/L)^a^	2.72(2.11,3.33)	2.70(2.13,3.32)	2.72(2.11,3.33)	0.436
*Prescription*				
The number of types of pills	5.28 ± 1.43	5.73 ± 1.20	5.03 ± 1.48	< 0.001

Values are n (%), unless otherwise specified. Data are presented as the mean ± SD if appropriate. *P* values were obtained with Student’s *t*-tests for continuous variables and chi-square test for categorical variables.

*BMI* body mass index, *PCI* percutaneous transluminal coronary intervention, *CABG* coronary artery bypass grafting, *MI* myocardial infarction, *CHF* chronic heart failure, *ACS* acute coronary syndrome, *PLT* platelets, *HGB* hemoglobin, *FBG* fasting blood glucose, *SCR* serum creatinine, *LDL-C* low-density lipoprotein cholesterol

^a^Data did not have a Gaussian distribution. *P* values were obtained with the Mann–Whitney *U* test

### Utilization of OMT and its components in patients after PCI

**Fig. 2:**
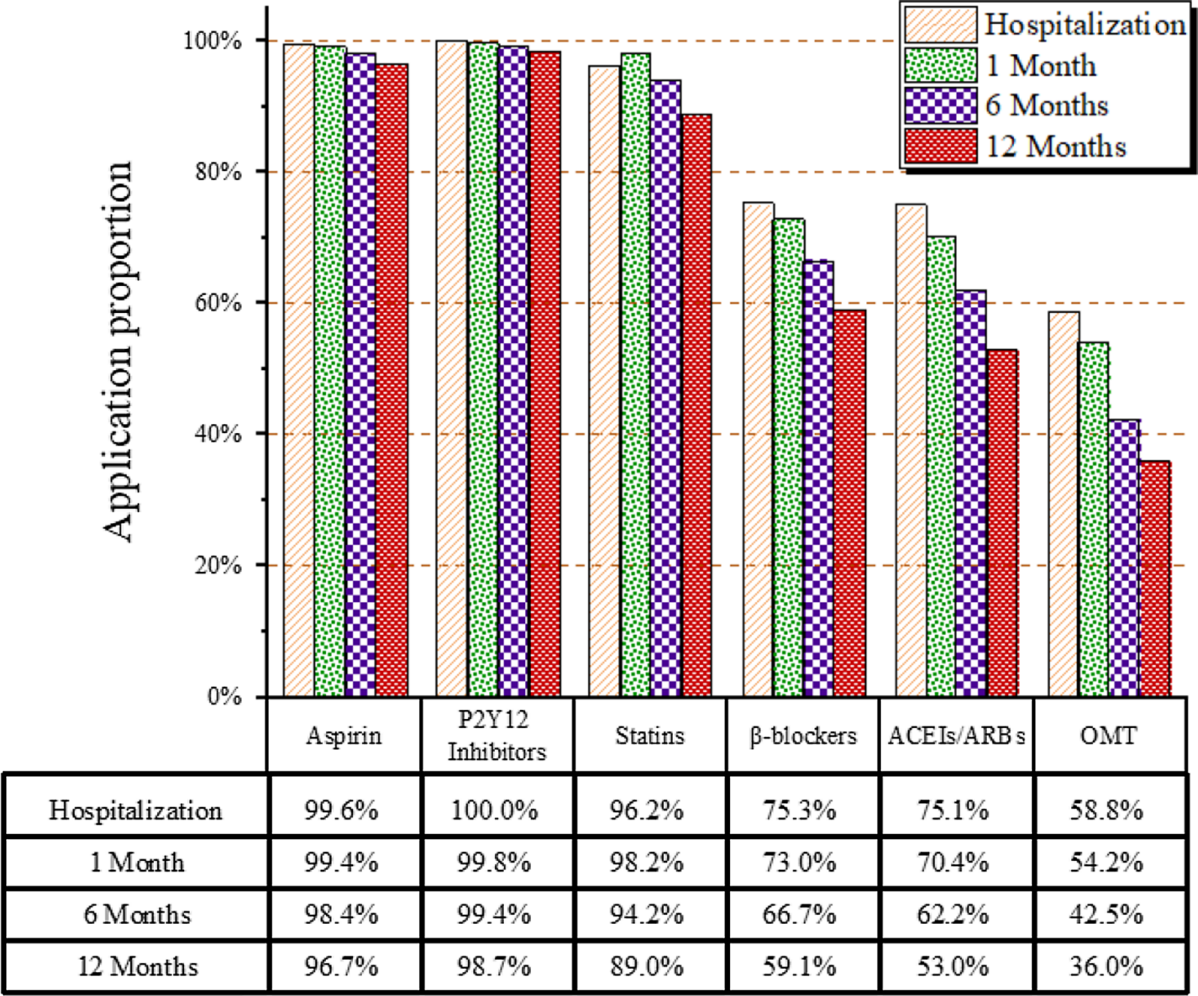
Trends in the utilization of OMT among eligible patients

Of the 3588 patients included in the final analysis, 58.8% received OMT during hospitalization. The utilization rates of aspirin tablets, P2Y12 receptor antagonists, statins, β-blockers and ACEI/ARB were 99.6%, 100%, 96.2%, 75.3 and 75.1%, respectively, in the hospital at baseline. As the time since discharge increased, the utilization rate of aspirin tablets, P2Y12 receptor antagonists, statins, β-blockers and ACEIs/ARBs gradually decreased (decrease to 97.0%, 98.7%, 88.9%, 59.4 and 53.0%, respectively, at the end of follow-up); this was especially notable for ACEIs/ARBs and β-blockers, which showed the most significant decreases. However, the utilization rate of antiplatelet drugs still exceeded 90%. The proportion of patients who remained adherent to OMT for 12 months was 36.0%, with a reduction of 38.7% over time (Fig. 2).

### Predictors of OMT status

As shown in Table 1, the single-factor analysis showed that age, BMI, medical insurance, education, history of PCI, comorbidities, number of coronary lesions, the number of types of pills, FBG, and SCR influenced OMT status after PCI. There were significant differences in OMT based on all the aforementioned variables (*P* < 0.05). After multivariable analysis (method: forward logistic regression), we found that the baseline OMT (OR = 52.868, 95% CI 38.129–73.305, *P* < 0.001) was a strong predictor of OMT after PCI, as shown in Table 2. The Hosmer–Lemeshow test *P *value was 0.581, indicating good analogue effects.

**Table 2:** Multivariable logistic regression analysis of the predictors of OMT status after PCI

Demographic and clinical factors		*P* value	OR	95% CI
Education		< 0.001		
	I VS P	0.060	0.800	0.634–1.010
	A VS P	0.001	1.435	1.157–1.780
Medical insurance		< 0.001		
	B VS *N*	0.001	1.594	1.216–2.088
	E VS *N*	< 0.001	2.730	2.097–3.555
Prior PCI	Y VS N	0.028	1.260	1.026–1.548
Hypertension	Y VS N	< 0.001	2.373	1.932–2.915
Hyperlipidaemia	Y VS N	0.008	1.274	1.065–1.524
Diabetes	Y VS N	0.001	1.395	1.148–1.696
The number of types of pills^a^		< 0.001	0.829	0.765–0.898
Baseline OMT	Y VS N	< 0.001	52.868	38.129–73.305

*I* intermediate, *P* primary, *A* advanced, *B* basic medical insurance, *E* employee medical insurance, *Y* yes, *N* no, *CI* confidence interval, *OR* odds ratio

^a^The B value in the multivariable logistic regression analysis is negative, indicating a negative correlation

### Correlation between MACCEs and adherence to OMT

**Fig. 3:**
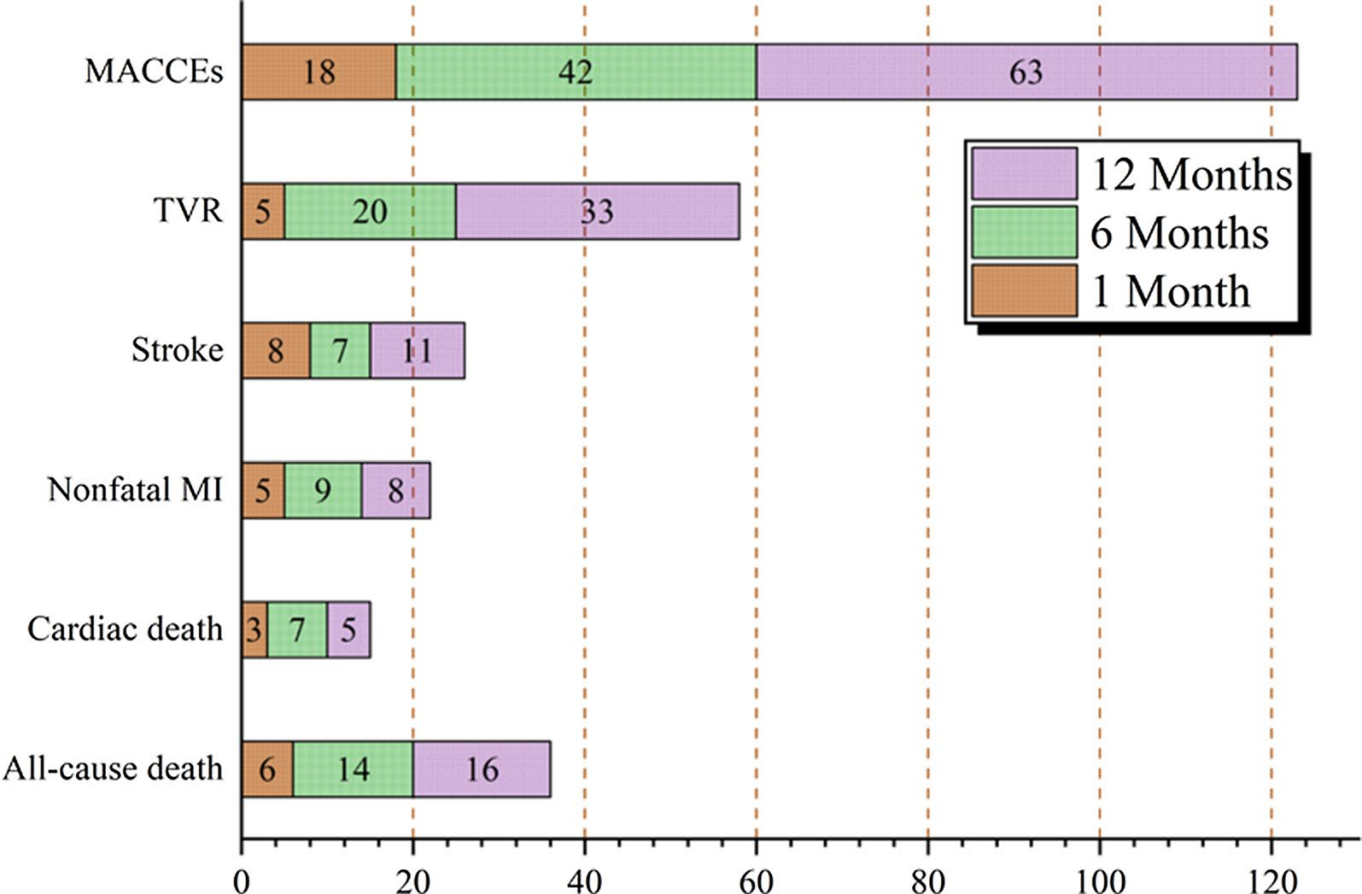
The occurrence of MACCEs in post-PCI patients

**Fig. 4:**
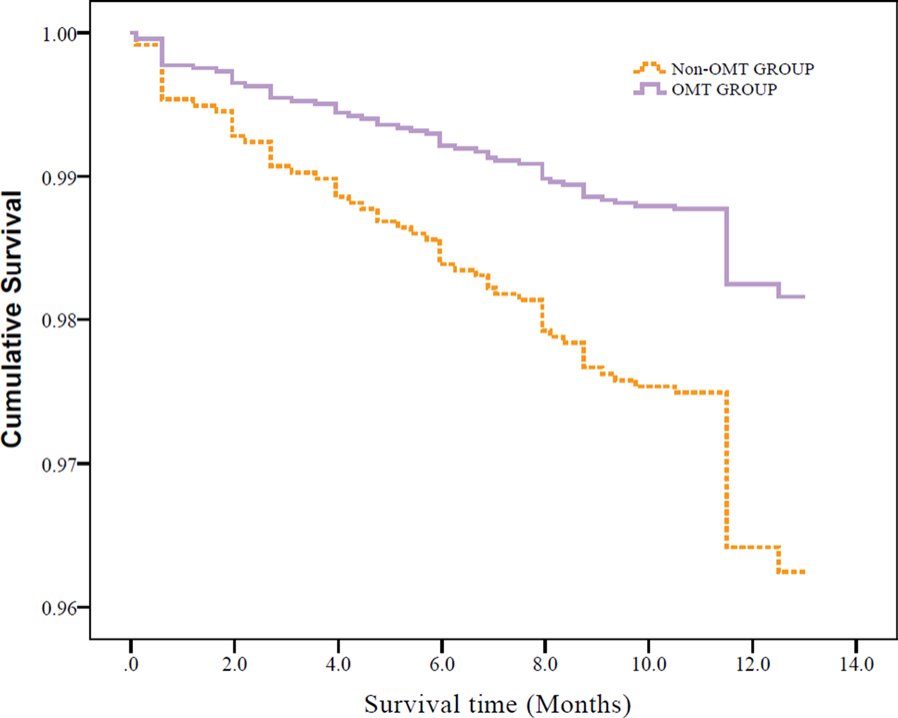
Event-free survival of MACCEs (in the OMT and non-OMT groups)

By the end of follow-up, MACCE occurred in 123 of 3588 patients (3.4%) after PCI. Among them, 36 patients died of all causes (15 patients died of cardiac death), 22 patients had nonfatal myocardial infarction, 26 patients had stroke, and 58 patients had TVRs (see Fig. 3). Propensity-score matching in a 1:1 ratio was used to balance the influence of baseline variables on OMT and eliminate confounders (matching factors included age, BMI, history of PCI, hypertension, hyperlipidemia, diabetes, number of coronary lesions, complications, FBG, and SCR), and 2598 patients were included in the final statistical analysis. A Cox hazard ratio model found that adherence to OMT at the 1-year follow-up (*P* = 0.001, *HR* = 0.486, 95% CI 0.312–0.756) was a significant prognostic factor for a lower incidence of MACCEs (see Table 3). The survival function curve based on the Cox regression model illustrated that the event-free survival of MACCEs was significantly higher in the OMT group than in the non-OMT group (Fig. 4). OMT adherence after PCI was a decisive protective factor against MACCEs.

**Table 3:** Multivariable Cox regression analysis of independent predictors of MACCEs in 1 year of follow-up

Variables	*P* value	*HR*	95% CI
Age ≥ 65 years	0.222	1.305	0.851–2.003
BMI (kg/m^2^)	0.071	0.937	0.873–1.005
ACS	0.912	0.965	0.510–1.825
History of MI	0.119	1.543	0.894–2.662
History of PCI	0.160	1.411	0.873–2.280
History of CRF	0.010	2.568	1.250–5.276
Number of comorbidities^a^	0.064	1.864	0.965–3.604
Hypertension	0.518	0.801	0.410–1.567
Diabetes	0.643	0.887	0.533–1.474
Hyperlipidemia	0.934	1.021	0.624–1.672
Multivessel lesions	0.043	1.887	1.021–3.485
Smoking	0.001	2.060	1.346–3.151
OMT (vs non-OMT)	0.001	0.486	0.312–0.756

Data were analyzed with a Cox regression model

*OMT* optimal medical treatment, *MI* myocardial infarction, *CI* confidence interval, *OR* odds ratio, *CRF* chronic renal failure

^a^HR for each increase in comorbidities compared to patients without comorbidities

## Discussion

To the best of our knowledge, this is the first of few large-sample studies to evaluate the correlation between adherence to OMT and the occurrence of MACCEs after PCI in Chinese patients and to investigate the clinical and social factors affecting compliance with OMT.

Our study drew some conclusions as follows: (1) There was still a gap between OMT utilization after PCI and the recommendations in the evidence-based guidelines. With increasing time since discharge, the utilization rates of the components of OMT gradually decreased, especially ACEIs/ARBs and β-blockers. The adherence to antiplatelets and statins at 1 year was generally good. However, we should pay more attention to the adherence to ACEIs/ARBs and β-blockers. (2) Education, type of medical insurance, baseline OMT status, number of types of pills, and the history of PCI, hypertension, hyperlipidemia, and diabetes influence OMT utilization in patients after PCI. (3) Smoking cessation after PCI is crucial for improving the prognosis of CHD patients. (4) Furthermore, after Cox multivariate adjustment, OMT is an independent protective factor against the incidence of MACCEs after PCI.

It is noteworthy that medical therapy is the foundation of the management of CHD. Clinical guidelines recommend the evidence-based use of OMT as secondary prevention to reduce the risk of events associated with CHD. Previous studies indicated that the utilization of OMT decreased as time passed after discharge, and risk factor control was generally suboptimal [13, 14]. Medication adherence was independently associated with a favorable prognosis [15–17]. Only 36% of the patients were still adherent to OMT at the end of follow-up. Our results are consistent with those of a previous trial, which noted that OMT was associated with a 51.4% reduction in the 1-year risk of MACCEs (*P* = 0.001, *HR* = 0.486, 95% CI 0.312–0.756). Iqbal et al. [16] reported that the most prominent effect of OMT was seen within the first year after revascularization because the majority of MACCEs occurred within the first year. In addition, the beneficial effect of OMT was sustained throughout the 5-year follow-up. He et al. [17] reported that the use of OMT was associated with fewer hospitalizations and lower all-cause direct medical costs among patients in China. OMT is significant to reduce the risk of future MACCEs.

Our study also found that smoking cessation after PCI is crucial. Smoking itself is a risk factor for CHD. Smoking cessation is beneficial for the secondary prevention of CHD. However, it is undeniable that there are still some patients who continue to smoke, which leads to higher rates of all-cause mortality and MACCEs, seriously affecting patient prognosis [18]. Smoking affects drug efficacy and is associated with a greater risk of high platelet reactivity [19]. In addition, smoking is related to various markers of inflammation that have been found to be independent risk factors for CHD [20]. Our study supports the finding that there are severe consequences of continuing to smoke after PCI, as doing so was associated with a more than 200% higher risk of MACCEs in the first year after PCI (*P* = 0.001, *HR* = 2.060, 95% CI 1.346–3.151).

The burden of CHD is very high in China due to the high prevalence and poor control of risk factors in the large Chinese population [21]. Yang et al. [22] reported that there are geographic differences in the management of CHD after PCI in China, and targeted adjustments of strategies with the consideration of these geographic differences are needed in the future to enhance CHD management in China as a whole and in specific provinces. Therefore, with coordination among the medical institutions within each region, a long-term follow-up strategy for patients with cardiovascular diseases is gradually being constructed to maintain their adherence to OMT and reduce the readmission rate, thereby improving patient health and promoting the rational use of social resources.

Adherence to OMT is a crucial factor affecting the progression and prognosis of CHD. The factors influencing OMT status after PCI were found to be multifaceted. The results of our study can be used by physicians to improve patient compliance with OMT after PCI. Physicians can use information (including sociodemographic data and clinical history) obtained during a patient’s hospitalization to predict that patient’s compliance with the medication regimen after discharge. Binary logistic regression analysis found that education, type of medical insurance, baseline OMT status, number of pill type, and history of hypertension, hyperlipidemia and diabetes influenced OMT application in patients after PCI. Baseline OMT status was the strongest predictor. The baseline OMT status reflected physician prescribing practices. Kähkönen et al. [23] reported that a prescription is a predictor at discharge of high postoperative OMT adherence by patients. The Get with the Guidelines project [24] (GWTG) in the United States advocated for increasing the prescription of OMT to ACS patients during hospitalization because it could increase the long-term out-of-hospital medication adherence among these patients. Our results showed that the education level affected OMT status. A higher level of education has been found to be associated with better patient awareness and cooperation with health care professionals [25]. Employee medical insurance has a higher reimbursement rate than basic medical insurance. Educationally or economically disadvantaged patients need more support and information. Another study found that first-time PCI patients underestimated their condition and lacked knowledge of their disease [26, 27]. Nursing after PCI should continue into the outpatient and community settings. In addition, general practitioners play an irreplaceable role in patient management and rehabilitation after PCI. We should establish a hospital-community-family CHD management model, with grading diagnosis and treatment performed by both specialists and general practitioners, and establish diagnostic and treatment norms for CHD in primary medical institutions. The number of types of drugs can be represented by the daily number of pills prescribed. Several studies have shown that clinicians can improve adherence by reducing patients’ daily pill burden when possible [28]. We can simplify patients’ prescriptions and use combination pills when available to improve adherence. This study has several potential implications for clinical practice. These predictors provide a basis for the use of OMT in patients after PCI. Based on the findings of this study, management methods can be formulated to increase the proportion of patients who remain adherent to OMT, reduce the incidence of MACCEs, reduce the rate of rehospitalization, reduce medical expenditures, and improve the health of post-PCI patients. OMT is a new method of managing chronic disease in primary hospitals.

Our present study has some limitations. First, our study was a single-center observational study, and our current findings may not be generalizable to other populations due to selection bias. Second, the follow-up time was relatively short, while the application of OMT after PCI continues in the long-term. It is expected that future studies with longer follow-up durations after PCI will help guide clinical practice. Third, the reasons for discontinuing drug treatment were not collected in this study design. Finally, although we extensively adjusted for some covariates, it remains possible that unknown confounders influenced the association between medication nonadherence and MACCEs and the factors influencing the utilization of OMT.

## Conclusions

Overall, the utilization of OMT after PCI is still inadequate compared with the recommendations in the evidence-based guidelines. The 1-year rates of adherence to antiplatelets and statins were generally good. However, the utilization of ACEIs/ARBs and β-blockers should receive more attention. Sociodemographic and clinical factors influenced the utilization of OMT, and we can use these factors to predict the population at risk of nonadherence to OMT. The use of OMT is an independent protective factor that can reduce the incidence of MACCEs after PCI. Therefore, we should use the identified predictors to strengthen adherence to OMT after PCI, thereby improving patient outcomes. In addition, smoking cessation after PCI should also be promoted.

## Data Availability

The datasets used or analyzed during the current study are available from the corresponding author on reasonable request.

## Abbreviations

OMT: Optimal medical therapy
MACCE: Main adverse cardiovascular and cerebrovascular event
CHD: Coronary heart disease
PCI: Percutaneous coronary intervention
CABG: Coronary artery bypass grafting
BMI: Body mass index
CHF: Chronic heart failure
COPD: Chronic obstructive pulmonary disease
DAPT: Dual antiplatelet therapy
ACEIs: Angiotensin-converting enzyme inhibitors
ARBs: Angiotensin receptor blockers
TVR: Target vessel revascularization
OR: Odds ratio
CI: Confidence interval
HR: Hazard ratio
MI: Myocardial infarction
ACS: Acute coronary syndrome
PLT: Platelets
HGB: Hemoglobin
FBG: Fasting blood glucose
SCR: Serum creatinine
LDL-C: Low-density lipoprotein cholesterol
GWTG: Get with the guidelines project
